# *cis*-*N* ^1^,*N* ^2^-Bis(2-hy­droxy­benzyl­idene)cyclo­hexane-1,2-diamine

**DOI:** 10.1107/S1600536811049038

**Published:** 2011-11-23

**Authors:** Ping Fan, Chunhua Ge, Xiangdong Zhang, Rui Zhang, Su Li

**Affiliations:** a College of Chemistry, Liaoning University, Shenyang, Liaoning 110036, People’s Republic of China

## Abstract

In the title compound, C_20_H_22_N_2_O_2_, the cyclo­hexane ring adopts a chair conformation and the two N atoms bonded to salicyl­idene groups are in *cis* positions. Both hy­droxy groups are involved in intra­molecular O—H⋯N hydrogen bonding and the two benzene rings form a dihedral angle of 60.5 (1)°.

## Related literature

For the crystal structure of *trans*-*N,N′*-bis­(salicyl­idene)-1,2-cyclo­hexa­nediamine, see: Cannadine *et al.* (1996 ▶); Liu *et al.* (1997 ▶), and for the crystal structures of its complexes, see: Khalaji *et al.* (2010 ▶); Man *et al.* (2008 ▶); Xu *et al.* (2009 ▶).
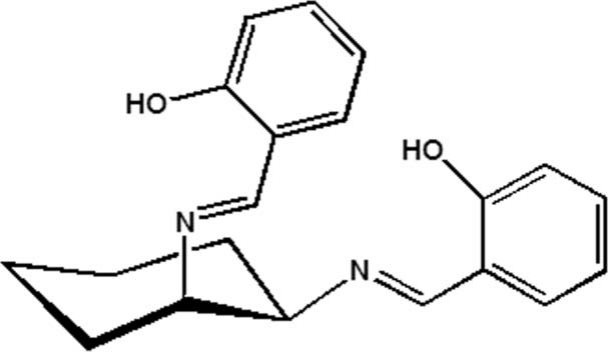


## Experimental

### 

#### Crystal data

C_20_H_22_N_2_O_2_
*M* *_r_* = 322.40
Orthorhombic, 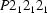

*a* = 6.125 (3) Å
*b* = 13.763 (6) Å
*c* = 21.537 (9) Å
*V* = 1815.4 (13) Å^3^
*Z* = 4
Mo *K*α radiation
μ = 0.08 mm^−1^
*T* = 296 K
0.25 × 0.15 × 0.12 mm

#### Data collection

Bruker SMART CCD area-detector diffractometer
Absorption correction: multi-scan (*SADABS*; Bruker, 2001 ▶) *T*_min_ = 0.987, *T*_max_ = 0.991
9439 measured reflections
4195 independent reflections
2178 reflections with *I* > 2σ(*I*)
*R*_int_ = 0.026

#### Refinement

*R*[*F* ^2^ > 2σ(*F* ^2^)] = 0.044
*wR*(*F* ^2^) = 0.115
*S* = 0.99
4195 reflections
219 parameters
H-atom parameters constrained
Δρ_max_ = 0.09 e Å^−3^
Δρ_min_ = −0.10 e Å^−3^

### 

Data collection: *SMART* (Bruker, 2001 ▶); cell refinement: *SAINT* (Bruker, 2001 ▶); data reduction: *SAINT*; program(s) used to solve structure: *SHELXS97* (Sheldrick, 2008 ▶); program(s) used to refine structure: *SHELXL97* (Sheldrick, 2008 ▶); molecular graphics: *SHELXTL* (Sheldrick, 2008 ▶); software used to prepare material for publication: *SHELXL97*, *PLATON* (Spek, 2009 ▶) and *WinGX* (Farrugia, 1999 ▶).

## Supplementary Material

Crystal structure: contains datablock(s) I, global. DOI: 10.1107/S1600536811049038/cv5199sup1.cif

Structure factors: contains datablock(s) I. DOI: 10.1107/S1600536811049038/cv5199Isup2.hkl

Supplementary material file. DOI: 10.1107/S1600536811049038/cv5199Isup3.cml

Additional supplementary materials:  crystallographic information; 3D view; checkCIF report

## Figures and Tables

**Table 1:** Hydrogen-bond geometry (Å, °)

*D*—H⋯*A*	*D*—H	H⋯*A*	*D*⋯*A*	*D*—H⋯*A*
O1—H1⋯N1	0.82	1.87	2.599 (3)	148
O2—H2⋯N2	0.82	1.84	2.577 (3)	148

**Fig. 1.**
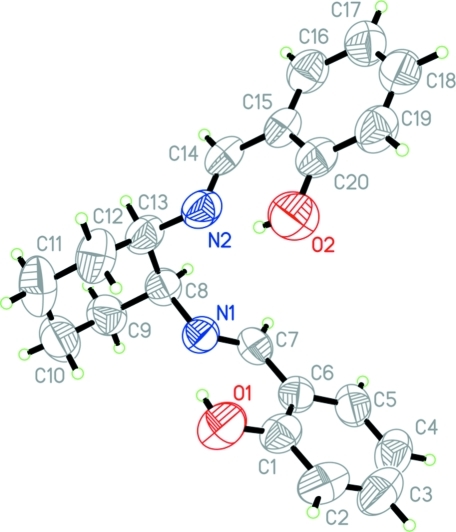
The molecular structure of the title compound. Displacement ellipsoids are drawn at the 50% probability level.

## supplementary crystallographic information

### Comment

1,2-Cyclohexanediamine has *trans-cis* isomers, so their reaction products are also isomers. *trans*-*N,N'*-Bis(salicylidene)-1,2-cyclohexanediamine has been prepared and characterized *via* X-ray crystallography (Cannadine *et al.*, 1996; Liu *et al.*, 1997). The compound is current of interest due to its fascinating versatility as coordination ligand (Khalaji *et al.*, 2010; Man *et al.*, 2008; Xu *et al.*, 2009). As *cis*-isomer is difficult to form complex, it has received relatively few studies.

The stucture of the title compound is shown in Fig. 1. The two *N*-salicylidene groups are *cis* in the structure with the same constitution but differ in the arrangement of their atoms in space. Dihedral angel between aromatic rings is 60.5 (1) °, between the ring of C1—C6 and C8/C10/C12 is 88.8 (1) °, between the ring of C15—C20 and C8/C10/C12 is 75.2 (1)°, respectively. hydroxy groups and imine groups are involved in intramolecular hydrogen bonding (Table 1).

### Experimental

The *cis-trans* mixture of 1,2-cyclohexanediamine was purchased from Alfa Aesar and was used as received without further purification. The title compound was obtained as following: added 0.05 mol salicylaldehyde slowly to ethanol solution of 1,2-cyclohexanediamine with stirring, then the resulting mixture was stirred 2 h under reflexing. By slow evaporation, yellow block-shape single crystals suitable for X-ray analysis were obtained within several days.

### Refinement

All H atoms were placed in geometrically idealized positions (C—H = 0.93 - 0.97 Å, and O—H = 0.82 Å), and refined in a riding model, with *U*_iso_(H) = 1.2-1.5 *U*_eq_ of the parent atom. In the absence of any significant anomalous scatterers in the molecule, the 1621 Friedel pairs were merged before the final refinement.

### Crystal data

**Table:**

C_20_H_22_N_2_O_2_	*F*(000) = 688
*M**_r_* = 322.40	*D*_x_ = 1.180 Mg m^−^^3^
Orthorhombic, *P*2_1_2_1_2_1_	Mo *K*α radiation, λ = 0.71073 Å
Hall symbol: P 2ac 2ab	Cell parameters from 126 reflections
*a* = 6.125 (3) Å	θ = 2.5–23.1°
*b* = 13.763 (6) Å	µ = 0.08 mm^−^^1^
*c* = 21.537 (9) Å	*T* = 296 K
*V* = 1815.4 (13) Å^3^	Block, yellow
*Z* = 4	0.25 × 0.15 × 0.12 mm

### Data collection

**Table:**

Bruker SMART CCD area-detector diffractometer	4195 independent reflections
Radiation source: fine-focus sealed tube	2178 reflections with *I* > 2σ(*I*)
graphite	*R*_int_ = 0.026
φ and ω scans	θ_max_ = 28.2°, θ_min_ = 1.9°
Absorption correction: multi-scan (*SADABS*; Bruker, 2001)	*h* = −8→8
*T*_min_ = 0.987, *T*_max_ = 0.991	*k* = −18→8
9439 measured reflections	*l* = −27→28

### Refinement

**Table:**

Refinement on *F*^2^	Primary atom site location: structure-invariant direct methods
Least-squares matrix: full	Secondary atom site location: difference Fourier map
*R*[*F*^2^ > 2σ(*F*^2^)] = 0.044	Hydrogen site location: inferred from neighbouring sites
*wR*(*F*^2^) = 0.115	H-atom parameters constrained
*S* = 0.99	*w* = 1/[σ^2^(*F*_o_^2^) + (0.0487*P*)^2^] where *P* = (*F*_o_^2^ + 2*F*_c_^2^)/3
4195 reflections	(Δ/σ)_max_ < 0.001
219 parameters	Δρ_max_ = 0.09 e Å^−^^3^
0 restraints	Δρ_min_ = −0.10 e Å^−^^3^

### Special details

**Table:**

Geometry. All e.s.d.'s (except the e.s.d. in the dihedral angle between two l.s. planes) are estimated using the full covariance matrix. The cell e.s.d.'s are taken into account individually in the estimation of e.s.d.'s in distances, angles and torsion angles; correlations between e.s.d.'s in cell parameters are only used when they are defined by crystal symmetry. An approximate (isotropic) treatment of cell e.s.d.'s is used for estimating e.s.d.'s involving l.s. planes.
Refinement. Refinement of *F*^2^ against ALL reflections. The weighted *R*-factor *wR* and goodness of fit *S* are based on *F*^2^, conventional *R*-factors *R* are based on *F*, with *F* set to zero for negative *F*^2^. The threshold expression of *F*^2^ > σ(*F*^2^) is used only for calculating *R*-factors(gt) *etc*. and is not relevant to the choice of reflections for refinement. *R*-factors based on *F*^2^ are statistically about twice as large as those based on *F*, and *R*- factors based on ALL data will be even larger.

### Fractional atomic coordinates and isotropic or equivalent isotropic displacement parameters (Å^2^)

**Table:**

	*x*	*y*	*z*	*U*_iso_*/*U*_eq_	
N2	−0.0052 (3)	1.09660 (12)	0.07839 (9)	0.0732 (5)	
N1	−0.0383 (3)	0.92191 (11)	0.14663 (8)	0.0654 (4)	
C14	−0.0964 (4)	1.13716 (14)	0.03205 (11)	0.0692 (6)	
H14	−0.2311	1.1672	0.0374	0.083*	
O1	0.3379 (3)	0.84294 (11)	0.17057 (9)	0.0955 (5)	
H1	0.2397	0.8833	0.1722	0.143*	
C15	0.0042 (3)	1.13768 (13)	−0.02904 (10)	0.0622 (5)	
C7	−0.0724 (4)	0.85231 (15)	0.10808 (9)	0.0642 (5)	
H7	−0.2020	0.8521	0.0856	0.077*	
C13	−0.1099 (4)	1.09759 (15)	0.13967 (10)	0.0736 (6)	
H13	−0.2294	1.1449	0.1388	0.088*	
C20	0.2131 (4)	1.09706 (15)	−0.03864 (11)	0.0699 (6)	
O2	0.3257 (3)	1.05667 (15)	0.00898 (8)	0.1061 (6)	
H2	0.2520	1.0593	0.0407	0.159*	
C8	−0.2060 (3)	0.99701 (14)	0.15462 (9)	0.0670 (6)	
H8	−0.3264	0.9838	0.1258	0.080*	
C16	−0.1018 (4)	1.17840 (16)	−0.07974 (12)	0.0803 (7)	
H16	−0.2379	1.2071	−0.0742	0.096*	
C5	0.0343 (4)	0.69815 (16)	0.05699 (9)	0.0795 (7)	
H5	−0.0973	0.6984	0.0355	0.095*	
C1	0.2831 (4)	0.77194 (15)	0.12906 (11)	0.0720 (6)	
C6	0.0818 (4)	0.77415 (14)	0.09806 (9)	0.0635 (6)	
C19	0.3026 (4)	1.09673 (16)	−0.09734 (12)	0.0848 (7)	
H19	0.4397	1.0695	−0.1038	0.102*	
C17	−0.0095 (5)	1.17723 (17)	−0.13826 (12)	0.0919 (8)	
H17	−0.0835	1.2042	−0.1718	0.110*	
C3	0.3740 (6)	0.62313 (19)	0.07855 (15)	0.1027 (9)	
H3	0.4721	0.5726	0.0719	0.123*	
C2	0.4287 (4)	0.69612 (18)	0.11904 (13)	0.0940 (8)	
H2A	0.5621	0.6949	0.1396	0.113*	
C18	0.1903 (5)	1.13633 (16)	−0.14639 (13)	0.0882 (7)	
H18	0.2521	1.1351	−0.1858	0.106*	
C9	−0.2941 (4)	0.99545 (17)	0.22072 (10)	0.0861 (7)	
H9A	−0.4196	1.0382	0.2234	0.103*	
H9B	−0.3427	0.9302	0.2306	0.103*	
C4	0.1800 (6)	0.62283 (17)	0.04797 (13)	0.0955 (9)	
H4	0.1454	0.5722	0.0211	0.115*	
C12	0.0551 (5)	1.13028 (19)	0.18700 (12)	0.1004 (8)	
H12A	0.1016	1.1960	0.1774	0.120*	
H12B	0.1822	1.0884	0.1850	0.120*	
C10	−0.1265 (5)	1.0268 (2)	0.26770 (11)	0.1056 (9)	
H10A	−0.0067	0.9807	0.2680	0.127*	
H10B	−0.1918	1.0272	0.3087	0.127*	
C11	−0.0396 (6)	1.1276 (2)	0.25261 (13)	0.1206 (10)	
H11A	0.0729	1.1451	0.2823	0.145*	
H11B	−0.1568	1.1747	0.2560	0.145*	

### Atomic displacement parameters (Å^2^)

**Table:**

	*U*^11^	*U*^22^	*U*^33^	*U*^12^	*U*^13^	*U*^23^
N2	0.0736 (12)	0.0638 (10)	0.0823 (12)	0.0124 (10)	0.0150 (11)	0.0022 (10)
N1	0.0665 (11)	0.0612 (10)	0.0686 (10)	0.0021 (9)	−0.0040 (9)	−0.0028 (9)
C14	0.0631 (14)	0.0491 (11)	0.0953 (16)	0.0074 (10)	0.0081 (14)	−0.0095 (11)
O1	0.0795 (11)	0.0822 (10)	0.1249 (12)	0.0047 (10)	−0.0239 (11)	−0.0169 (10)
C15	0.0558 (12)	0.0503 (11)	0.0807 (14)	−0.0014 (10)	0.0059 (12)	−0.0102 (10)
C7	0.0657 (14)	0.0729 (13)	0.0540 (11)	−0.0063 (12)	0.0026 (11)	0.0031 (11)
C13	0.0736 (15)	0.0627 (12)	0.0846 (15)	0.0136 (11)	0.0117 (14)	−0.0044 (12)
C20	0.0635 (14)	0.0615 (12)	0.0848 (16)	0.0066 (11)	0.0056 (14)	−0.0014 (12)
O2	0.0858 (12)	0.1315 (14)	0.1009 (12)	0.0447 (11)	0.0156 (11)	0.0126 (12)
C8	0.0585 (13)	0.0764 (13)	0.0662 (13)	0.0082 (12)	−0.0002 (11)	−0.0029 (11)
C16	0.0686 (15)	0.0780 (14)	0.0943 (17)	0.0114 (12)	−0.0031 (15)	−0.0093 (14)
C5	0.1023 (19)	0.0715 (14)	0.0649 (13)	−0.0134 (15)	0.0107 (14)	−0.0049 (11)
C1	0.0746 (16)	0.0562 (12)	0.0850 (16)	−0.0052 (12)	0.0086 (14)	0.0037 (12)
C6	0.0739 (16)	0.0567 (12)	0.0600 (12)	−0.0067 (12)	0.0075 (12)	0.0038 (10)
C19	0.0761 (16)	0.0802 (15)	0.0980 (18)	0.0085 (14)	0.0251 (16)	−0.0053 (14)
C17	0.104 (2)	0.0924 (17)	0.0795 (17)	0.0148 (16)	−0.0074 (17)	−0.0083 (14)
C3	0.116 (3)	0.0641 (16)	0.128 (2)	0.0091 (17)	0.046 (2)	0.0023 (16)
C2	0.0843 (18)	0.0730 (15)	0.125 (2)	0.0094 (15)	0.0115 (17)	0.0144 (16)
C18	0.104 (2)	0.0741 (15)	0.0870 (18)	0.0034 (16)	0.0185 (17)	−0.0029 (13)
C9	0.0863 (17)	0.0986 (16)	0.0734 (15)	0.0124 (15)	0.0108 (14)	0.0039 (13)
C4	0.133 (3)	0.0620 (15)	0.0916 (19)	−0.0111 (18)	0.037 (2)	−0.0087 (13)
C12	0.101 (2)	0.0944 (18)	0.1055 (19)	−0.0143 (16)	0.0050 (17)	−0.0323 (14)
C10	0.115 (2)	0.133 (2)	0.0689 (15)	0.028 (2)	−0.0044 (17)	−0.0073 (16)
C11	0.122 (2)	0.145 (3)	0.0947 (19)	−0.005 (2)	−0.0084 (19)	−0.0495 (19)

### Geometric parameters (Å, °)

**Table:**

N2—C14	1.273 (3)	C5—H5	0.9300
N2—C13	1.467 (3)	C1—C2	1.390 (3)
N1—C7	1.285 (2)	C1—C6	1.402 (3)
N1—C8	1.467 (2)	C19—C18	1.373 (3)
C14—C15	1.453 (3)	C19—H19	0.9300
C14—H14	0.9300	C17—C18	1.359 (4)
O1—C1	1.366 (2)	C17—H17	0.9300
O1—H1	0.8200	C3—C4	1.358 (4)
C15—C16	1.389 (3)	C3—C2	1.372 (4)
C15—C20	1.411 (3)	C3—H3	0.9300
C7—C6	1.448 (3)	C2—H2A	0.9300
C7—H7	0.9300	C18—H18	0.9300
C13—C12	1.504 (3)	C9—C10	1.504 (4)
C13—C8	1.538 (3)	C9—H9A	0.9700
C13—H13	0.9800	C9—H9B	0.9700
C20—O2	1.355 (2)	C4—H4	0.9300
C20—C19	1.378 (3)	C12—C11	1.528 (4)
O2—H2	0.8200	C12—H12A	0.9700
C8—C9	1.523 (3)	C12—H12B	0.9700
C8—H8	0.9800	C10—C11	1.522 (4)
C16—C17	1.382 (3)	C10—H10A	0.9700
C16—H16	0.9300	C10—H10B	0.9700
C5—C4	1.381 (4)	C11—H11A	0.9700
C5—C6	1.400 (3)	C11—H11B	0.9700
			
C14—N2—C13	120.62 (18)	C18—C19—H19	119.8
C7—N1—C8	119.17 (18)	C20—C19—H19	119.8
N2—C14—C15	121.72 (19)	C18—C17—C16	119.4 (3)
N2—C14—H14	119.1	C18—C17—H17	120.3
C15—C14—H14	119.1	C16—C17—H17	120.3
C1—O1—H1	109.5	C4—C3—C2	121.6 (3)
C16—C15—C20	118.0 (2)	C4—C3—H3	119.2
C16—C15—C14	121.0 (2)	C2—C3—H3	119.2
C20—C15—C14	121.0 (2)	C3—C2—C1	119.5 (3)
N1—C7—C6	123.0 (2)	C3—C2—H2A	120.3
N1—C7—H7	118.5	C1—C2—H2A	120.3
C6—C7—H7	118.5	C17—C18—C19	121.1 (2)
N2—C13—C12	108.59 (19)	C17—C18—H18	119.5
N2—C13—C8	110.31 (16)	C19—C18—H18	119.5
C12—C13—C8	112.61 (19)	C10—C9—C8	112.5 (2)
N2—C13—H13	108.4	C10—C9—H9A	109.1
C12—C13—H13	108.4	C8—C9—H9A	109.1
C8—C13—H13	108.4	C10—C9—H9B	109.1
O2—C20—C19	119.4 (2)	C8—C9—H9B	109.1
O2—C20—C15	120.8 (2)	H9A—C9—H9B	107.8
C19—C20—C15	119.7 (2)	C3—C4—C5	119.6 (3)
C20—O2—H2	109.5	C3—C4—H4	120.2
N1—C8—C9	110.34 (17)	C5—C4—H4	120.2
N1—C8—C13	109.95 (16)	C13—C12—C11	111.4 (2)
C9—C8—C13	110.13 (17)	C13—C12—H12A	109.3
N1—C8—H8	108.8	C11—C12—H12A	109.3
C9—C8—H8	108.8	C13—C12—H12B	109.3
C13—C8—H8	108.8	C11—C12—H12B	109.3
C17—C16—C15	121.4 (2)	H12A—C12—H12B	108.0
C17—C16—H16	119.3	C9—C10—C11	110.9 (2)
C15—C16—H16	119.3	C9—C10—H10A	109.5
C4—C5—C6	121.0 (3)	C11—C10—H10A	109.5
C4—C5—H5	119.5	C9—C10—H10B	109.5
C6—C5—H5	119.5	C11—C10—H10B	109.5
O1—C1—C2	118.7 (2)	H10A—C10—H10B	108.1
O1—C1—C6	120.8 (2)	C10—C11—C12	110.6 (2)
C2—C1—C6	120.4 (2)	C10—C11—H11A	109.5
C5—C6—C1	117.9 (2)	C12—C11—H11A	109.5
C5—C6—C7	120.9 (2)	C10—C11—H11B	109.5
C1—C6—C7	121.2 (2)	C12—C11—H11B	109.5
C18—C19—C20	120.4 (2)	H11A—C11—H11B	108.1

### Hydrogen-bond geometry (Å, °)

**Table:**

*D*—H···*A*	*D*—H	H···*A*	*D*···*A*	*D*—H···*A*
O1—H1···N1	0.82	1.87	2.599 (3)	148
O2—H2···N2	0.82	1.84	2.577 (3)	148
